# No evidence of false-negative *Plasmodium falciparum* rapid diagnostic results in Monrovia, Liberia

**DOI:** 10.1186/s12936-021-03774-3

**Published:** 2021-05-26

**Authors:** Mandella King, Alexander E. George, Pau Cisteró, Christine K. Tarr-Attia, Beatriz Arregui, Senga Omeonga, Haily Chen, Ana Meyer García-Sípido, Adelaida Sarukhan, Quique Bassat, Dawoh Peter Lansana, Alfredo Mayor

**Affiliations:** 1 Saint Joseph’s Catholic Hospital, Tubman Boulevard, Oldest Congo, Town, PO Box 10512, 1100 Monrovia, Liberia; 2 Liberia Medicines & Health Products Regulatory Authority, VP Road, Old Road, Sinkor , PO Box 1994, Monrovia, Liberia; 3 ISGlobal, Barcelona Institute for Global Health, Hospital Clínic – Universitat de Barcelona, Carrer Rosselló 153 (CEK Building), 08036 Barcelona, Spain; 4 NGO Juan Ciudad Foundation, Madrid, Spain; 5 Centro de Investigação em Saúde de Manhiça (CISM), Maputo, Mozambique; 6 Consorcio de Investigación Biomédica en Red de Epidemiología y Salud Pública (CIBERESP), Madrid, Spain; 7 ICREA, Pg. Lluís Companys 23, 08010 Barcelona, Spain; 8 Pediatrics Department, Hospital Sant Joan de Déu, Universitat de Barcelona, Esplugues,, Barcelona, Spain

**Keywords:** Malaria, Liberia, Diagnostics, Microscopy, Rapid diagnostic tests, *pfhrp2* deletion

## Abstract

**Background:**

Malaria diagnosis in many malaria-endemic countries relies mainly on the use of rapid diagnostic tests (RDTs). The majority of commercial RDTs used in Africa detect the *Plasmodium falciparum* histidine-rich protein 2 (PfHRP2). *pfhrp2/3* gene deletions can therefore lead to false-negative RDT results. This study aimed to evaluate the frequency of PCR-confirmed, false-negative *P. falciparum* RDT results in Monrovia, Liberia.

**Methods:**

PfHRP2-based RDT (Paracheck Pf®) and microscopy results from 1038 individuals with fever or history of fever (n = 951) and pregnant women at first antenatal care (ANC) visit (n = 87) enrolled in the Saint Joseph’s Catholic Hospital (Monrovia) from March to July 2019 were used to assess the frequency of false-negative RDT results. True–false negatives were confirmed by detecting the presence of *P. falciparum* DNA by quantitative PCR in samples from individuals with discrepant RDT and microscopy results. Samples that were positive by 18S rRNA qPCR but negative by PfHRP2-RDT were subjected to multiplex qPCR assay for detection of *pfhrp2* and *pfhrp3*.

**Results:**

One-hundred and eighty-six (19.6%) and 200 (21.0%) of the 951 febrile participants had a *P. falciparum*-positive result by RDT and microscopy, respectively. Positivity rate increased with age and the reporting of joint pain, chills and shivers, vomiting and weakness, and decreased with the presence of coughs and nausea. The positivity rate at first ANC visit was 5.7% (n = 5) and 8% (n = 7) by RDT and microscopy, respectively. Out of 207 *Plasmodium* infections detected by microscopy, 22 (11%) were negative by RDT. qPCR confirmed absence of *P. falciparum* DNA in the 16 RDT-negative but microscopy-positive samples which were available for molecular testing. Among the 14 samples that were positive by qPCR but negative by RDT and microscopy, 3 only amplified *pfldh*, and among these 3 all were positive for *pfhrp2* and *pfhrp3*.

**Conclusion:**

There is no qPCR-confirmed evidence of false-negative RDT results due to *pfhrp2/pfhrp3* deletions in this study conducted in Monrovia (Liberia). This indicates that these deletions are not expected to affect the performance of PfHRP2-based RDTs for the diagnosis of malaria in Liberia. Nevertheless, active surveillance for the emergence of PfHRP2 deletions is required.

## Background

Delay in diagnosis and treatment is a leading cause of death in malaria patients [1]. The recommendation issued in 2010 by the World Health Organization (WHO) to restrict malaria treatment to parasitological confirmed malaria infections has boosted the use of rapid diagnostic tests (RDTs), which have now become a critical component of management and surveillance of malaria. Indeed, it has been estimated that, in 2019, over 348 million RDTs were sold by manufacturers and 267 million distributed by national malaria programmes, at a cost of hundreds of millions of euros [2].

Most RDTs manufactured, purchased and used around the world are based on the detection of *Plasmodium falciparum* histidine-rich protein 2 (PfHRP2), alone or in combination with other antigens (*Plasmodium* lactate dehydrogenase (pLDH) and *Plasmodium* aldolase (pAldo)). The PfHRP2 is a parasite-specific protein produced only by *P. falciparum* (and not the other human malaria parasite species) throughout its asexual life cycle, and released during schizogony into the peripheral circulation [3], where it can persist for weeks after the elimination of parasites [4]. In 2010, it was shown that some isolates of *P. falciparum* in Peru lacked the *pfhrp2* gene [5]. The *pfhrp3* gene is highly homologous to *pfhrp2* [6], and parasites lacking both *pfhrp2* and *pfhrp3* genes, or substantial parts of these genes, do not express functional proteins and are therefore not detected by PfHRP2-based RDTs [7]. Such false negative results pose a serious threat to case management, as patients truly infected with *P. falciparum* may be falsely identified as malaria-free, and thus not managed adequately. Recently, numerous studies have reported *P. falciparum* parasites lacking *pfhrp2* and *pfhrp3* genes in several countries in Africa [8–16], with *pfhrp2* deletion having been identified by WHO as one of the biological challenges currently threatening malaria control and elimination efforts. A mathematical model identified that a low intensity of transmission and a high frequency of treatment based on RDT detection of infection are the two main drivers of selection of *pfhrp2*-deleted parasites [17]. Current WHO recommendations suggest switching to non-PfHRP2 RDTs when the prevalence of *pfhrp2*-deleted parasites reaches the lower 90% confidence interval for 5% prevalence, or a plan for change if deletions surpass a frequency of 5% [18]. Good quality data are required to guide this switch, particularly taking into account that non-PfHRP2-based RDTs can have lower sensitivity than those based on the detection of PfHRP2 [19]. Systematic monitoring of parasites with *pfhrp2/3* deletions is therefore required to monitor the risk of false-negative RDT results.

RDT-negative but microscopy-positive results can occur due to operator error, inappropriate storage, limited performance of specific RDT brands and lots, low-parasite density infections, and *pfhrp2/pfhrp3* deletions. Mutant parasites carrying the deletion are usually identified by a discrepancy between positive microscopy results and negative results of the PfHRP2-based RDT in patients undergoing both tests [5, 20]. The detection of parasite DNA by polymerase chain reaction (PCR) offers the possibility of detecting low density infections that are not readily detected by RDT and the genomic confirmation of complete or partial deletions of the *pfhrp2/3* gene [7]. This study aimed to assess the frequency of true (PCR-confirmed) false-negative *P. falciparum Pf*HRP2 RDT results among symptomatic patients and pregnant women at first antenatal care (ANC) visit attending a public hospital in Monrovia, Liberia.

## Methods

### Study site and population

The study was conducted at the Outpatients Department, Emergency and Antenatal Consultation of the not-for-profit Saint Joseph’s Catholic Hospital (SJCH) in Congo Town neighbourhood, Monrovia. The SJCH provides general services to the population in Monrovia. Although the SJCH applies a cost recovery system for the general public, the institution has a charity arm to subsidize healthcare-related costs for the most deprived.

In the time-period 21 March to 21 July 2019, all patients who presented at the facilities with fever (temperature ≥ 37.5 °C) or history of fever during the preceding week, as well as pregnant women attending ANC for the first time during their pregnancy (irrespective of their fever status), were eligible for inclusion in the study. No individuals meeting inclusion criteria were excluded based on their race, social or economic status, religion, ethnic affiliation, nationality, political affiliation, or sexual orientation.

### Recruitment

Eligible patients were invited to participate and informed of the study objectives and specimen collection procedures. After providing written informed consent, they were queried on basic socio-demographic and malaria prevention-related data. Their forehead temperature was measured with an infrared thermometer. For the participants attending their first ANC, the gestational age was assessed by date of last menstrual period and by measurement of fundal height. Data were manually captured by the recruiting research team using individual standardized paper-based case report forms.

### Parasitological assessments

Participants were finger pricked for malaria testing. Five μl of blood were used to perform malaria testing using Paracheck Pf® (Orchid Biomedical Systems, Goa, India), a PfHRP2-based malaria RDT. Another 5 μl of blood was used to prepare a thick blood film for malaria parasite microscopy examination. If present, malaria parasites were detected and semi-quantified through the microscopic examination of Field’s stained thick blood film. A total of 9 technicians were in charge of the microscopic diagnosis. The diagnosis was made by a single technician without double confirmation. In case of any doubt, the expert opinion of the laboratory manager was used for a final decision. A 50-μl blood drop was spotted on Whatman 903 filter papers, dried for 24 h and stored in plastic bags with silica gel at -20ºC. One of the prepared filter papers was shipped to the Barcelona Institute of Global Health (Barcelona, Spain) for molecular detection of *P. falciparum*. All the samples with microscopy-, and RDT-discrepant results, plus a 25% random selection of the rest of samples, were selected for molecular assessment. DNA was extracted from filter papers following the Chelex method [21] and used for quantitative real-time PCR targeting *P. falciparum* 18S ribosomal RNA (rRNA) gene [21, 22]. In brief, purified DNA templates were amplified in an ABI PRISM 7500 Real-Time System (Applied Biosystems) following a previously described method [13]. Briefly, a 20-μl PCR mixture was performed using 5 μl of template, 10 μl of 2 × TaqMan® Universal PCR Master Mix (Applied Biosystems), a 300-nM concentration of each primers specific for 18S rRNA gene of *P. falciparum*, and a 150-nM concentration of probe labelled with 6-carboxy-fluorescein (FAM) as a reporter and 6-carboxytetramethylrhodamine (TAMRA) as a quencher. Amplification and detection were performed under the following conditions: 2 min at 50 °C, 10 min at 95 °C, and 40 cycles of 15 s at 95 °C and 1 min at 60 °C. The results were automatically analysed by the ABI Prism SDS2.0.6 software. Each specimen was run in duplicate. Parasitaemia was calculated by extrapolation against a standard curve of 5 serially diluted points prepared with known numbers of 3D7 ring-infected erythrocytes [23]. Samples without amplification (no Ct detected) were considered negative. A negative control (uninfected erythrocytes) and a blank control (no template) were run in all reactions.

Samples positive by microscopy and negative by the 18S *P. falciparum* qPCR were analysed by qPCR for *Plasmodium* spp using one set of generic primers targeting a highly conserved region of the 18S rRNA gene of the genus *Plasmodium* [24]. Amplification and detection of the amplified product were performed on an ABI Prism 7500 (Applied Biosystems). A negative control (uninfected erythrocytes) and a blank control (no template) as well as 4 positive controls (one for *P. falciparum, P. vivax, P. ovale*, and *P. malariae*) were included. The sample was considered positive by identifying the threshold cycle number (Ct) at which normalized reporter dye emission raised above background noise. If the fluorescent signal did not increase within 40 cycles (Ct = 40), the sample was considered negative.

Samples that were positive by 18S rRNA qPCR but negative by PfHRP2-RDT were subjected to multiplex qPCR assay for detection of *pfhrp2* and *pfhrp3* following previously described method [25] with minor modifications. In brief, an ABI 7500 real-time machine was used, with the following QSY probes (Applied Biosystems): Pfhrp2_probe 6FAM 5′-ATGCAAAAGGACTTAATTTAAATAAGAGATT-3′; Pfhrp3_probe VIC 5′-ACAATTCCCATACTTTACATCATGCA-3′; Pfldh_probe ABY 5′-GTAATAGTAACAGCTGGATTTACCAAGGCCCCA-3′; HumanTuBB_P JUN 5′-TTAACGTGCAGAACAAGAACAGCAGCT-3′.

To optimize the final reaction, *pfhrp2* primers and probe were increased to 900 nM and 250 nM, respectively. TaqMan® Multiplex Master Mix was used. The thermocycling conditions were 20 s at 95 °C, followed by 45 cycles of 3 s at 95 °C and 33 s at 60 °C. Samples with *HumTuBB* positive and *pfldh* positive but negative for *pfhrp2* or *pfhrp3* were considered as *pfhrp2*-deleted and *pfhrp3*-deleted, respectively. Samples with *HumTuBB* positive but *pfldh* negative were defined as having a parasitaemia too low to be detected by this method. Finally, samples with a *HumTuBB* negative result were considered to be invalid and indicated the need to repeat the DNA extraction and/or PCR experiment.

### Data management and statistical analysis

The study participants were assigned with a sequential unique identification number (UIN) that linked the signed consent forms to the case report forms. The case report forms did not include personal identifiers and were used to collect socio-demographic and malaria care-related data. The laboratory technologists were oriented to document all laboratory test results and report both the blood film and RDT results in standard reporting forms. All information contained in the case report forms was double-captured (by a trained laboratory technician and by a member of the research team) into a research database built in Microsoft Excel. The point-of-access to the Excel spreadsheets were designed to protect the confidentiality and integrity of the data and included authorization, authentication, auditing, and availability features to safeguard the access and usage of the data. The Excel spreadsheets were in a password-protected computer sited at the SJCH laboratory.

Data entered into Excel was converted to STATA (version 8.0, STATA Corporation, College Station, TX, USA) for further analyses. The sensitivity, specificity, false negative, and false positive values of Paracheck Pf® were calculated using microscopy as the gold standard. Briefly, sensitivity was calculated as the proportion of positive test results against true positives; specificity was calculated as a proportion of negative test results against true negatives. Negative RDT results were considered false-negatives if microscopy result was positive. Positive RDTs were considered false-positives if microscopy was negative. True false-negatives and false-positives were considered if the qPCR was positive and negative, respectively. Proportions were compared using Chi2 test and differences with a probability of less than 0.05 (P < 0.05) were accepted as significant.

## Results

### Clinical and demographic characteristics of study participants

One-thousand and forty participants meeting inclusion criteria were invited to participate between 21 March and 21 July, 2019, all of whom consented. Of the 1040 enrolled participants, two discontinued their participation before blood specimen collection. The analysis presented is based on data from 1038 participants (951 febrile individuals and 87 pregnant women) with peripheral blood samples available and analysed for *P. falciparum* infection. From the 951 febrile participants (Table 1), 459 were males (48%), 330 (35%) under 5 years of age, 387 (41%) above 20 years, and 541 (57%) reported primary education or below. Use of insecticide-treated nets and household indoor residual spraying was reported by 441 (46%) and 532 (56%) of the study participants, respectively. Three-hundred and sixty-five (38%) presented with fever and 725 (76%) reported fever during the preceding week. Malaria signs and symptoms ranged from 13% in the case of diarrhoea (n = 119) to 44% in the case of headache (n = 420). Seven-hundred and six of the study participants (74%) reported history of a previous malaria episode.

**Table 1:** *Plasmodium falciparum* positivity rates among the individuals with fever or history of fever during the previous week, by demographic and clinical variables

	RDT					Microscopy					PCR				
	Neg		Pos			Neg		Pos			Neg		Pos		
	n = 765		n = 186			n = 751		n = 200			n = 185		n = 77		
	n	%	n	%	p	n	%	n	%	p	n	%	n	%	p
Gender															
Female	398	52	93	50	0.624	390	52	101	51	0.750	98	53	45	58	0.496
Male	366	48	93	50		360	48	99	50		87	47	32	42	
Age (in years)															
< 5	308	40	22	12	** < 0.001**	304	40	26	13	** < 0.001**	37	20	10	13	0.142
5–10	98	13	15	8		91	12	22	11		36	19	9	12	
10–20	64	8	57	31		62	8	59	30		36	19	21	27	
> 20	295	39	92	49		294	39	93	47		76	41	37	48	
Previous malaria															
No	209	27	36	19	**0.025**	201	27	44	22	0.203	51	28	15	19	0.212
Yes	556	73	150	81		550	73	156	78		134	72	62	81	
ITN															
No	407	53	103	55	0.623	402	54	108	54	0.936	105	57	43	56	0.892
Yes	358	47	83	45		349	46	92	46		80	43	34	44	
IRS															
No	329	43	90	48	0.189	323	43	96	48	0.229	84	45	37	48	0.786
Yes	436	57	96	52		428	57	104	52		101	55	40	52	
Fever															
No	477	62	109	59	0.356	466	62	120	60	0.624	130	70	40	52	**0.007**
Yes	288	38	77	41		285	38	80	40		55	30	37	48	
History fever															
No	186	24	40	22	0.444	183	24	43	22	0.455	35	19	20	26	0.244
Yes	579	76	146	78		568	76	157	79		150	81	57	74	
Abdominal pain															
No	627	82	144	77	0.175	616	82	155	78	0.155	147	79	56	73	0.257
Yes	138	18	42	23		135	18	45	23		38	21	21	27	
Joint pain															
No	617	81	116	62	** < 0.001**	604	80	129	65	** < 0.001**	156	84	48	62	** < 0.001**
Yes	148	19	70	38		147	20	71	36		29	16	29	38	
Body ache															
No	669	87	158	85	0.395	659	88	168	84	0.193	154	83	61	79	0.481
Yes	96	13	28	15		92	12	32	16		31	17	16	21	
Cough															
No	437	57	136	73	** < 0.001**	432	58	141	71	**0.001**	121	65	64	83	**0.005**
Yes	328	43	50	27		319	42	59	30		64	35	13	17	
Diarrhoea															
No	675	88	157	84	0.174	660	88	172	86	0.472	163	88	64	83	0.320
Yes	90	12	29	16		91	12	28	14		22	12	13	17	
Headache															
No	465	61	66	35	** < 0.001**	454	60	77	39	** < 0.001**	111	60	30	39	**0.003**
Yes	300	39	120	65		297	40	123	62		74	40	47	61	
Nausea															
No	655	86	170	91	**0.040**	642	85	183	92	**0.026**	172	93	69	90	0.453
Yes	110	14	16	9		109	15	17	9		13	7	8	10	
Vomiting															
No	608	79	128	69	**0.002**	596	79	140	70	**0.006**	157	85	58	75	0.078
Yes	157	21	58	31		155	21	60	30		28	15	19	25	
Sweat															
No	639	84	156	84	1.000	628	84	167	84	1.000	162	88	66	86	0.690
Yes	126	16	30	16		123	16	33	17		23	12	11	14	
Chills and shivers															
No	591	77	124	67	**0.003**	581	77	134	67	**0.003**	152	82	59	77	0.308
Yes	174	23	62	33		170	23	66	33		33	18	18	23	
Weakness															
No	494	65	76	41	** < 0.001**	483	64	87	44	** < 0.001**	134	72	30	39	** < 0.001**
Yes	271	35	110	59		268	36	113	57		51	28	47	61	

RDT: Rapid diagnostic test; ITN: Insecticide treated nets; IRS: indoor residual spraying

Out of the 87 women at first ANC visit, 4 (5%) and 7 (8%) had fever or reported any sign/symptom of malaria at their first ANC visit, respectively (Table 2).

**Table 2:** *Plasmodium falciparum* positivity rates among pregnant women at first antenatal visit, by demographic and clinical variables

	RDT					Microscopy					PCR				
	Neg		Pos			Neg		Pos			Neg		Pos		
	n = 82		n = 5			n = 80		n = 7			n = 50		n = 9		
	n	%	n	%	p	n	%	n	%	p	n	%	n	%	p
Age (in years)															
10–20	15	18	1	20	1.000	15	19	1	14	1.000	9	18	1	11	1.000
> 20	67	82	4	80		65	81	6	86		41	82	8	89	
Previous malaria															
No	6	7	0	0	1.000	6	8	0	0	1.000	5	10	0	0	1.000
Yes	76	93	5	100		74	93	7	100		45	90	9	100	
ITN															
No	34	41	2	40	1.000	33	41	3	43	1.000	19	38	6	67	0.149
Yes	48	59	3	60		47	59	4	57		31	62	3	33	
IRS															
No	45	55	3	60	1.000	43	54	5	71	0.452	22	44	5	56	0.719
Yes	37	45	2	40		37	46	2	29		28	56	4	44	
Fever															
No	78	95	5	100	1.000	76	95	7	100	1.000	46	92	9	100	1.000
Yes	4	5	0	0		4	5	0	0		4	8	0	0	
History fever															
No	75	91	5	100	1.000	73	91	7	100	1.000	48	96	9	100	1.000
Yes	7	9	0	0		7	9	0	0		2	4	0	0	
Abdominal pain															
No	80	98	5	100	1.000	78	98	7	100	1.000	48	96	9	100	1.000
Yes	2	2	0	0		2	3	0	0		2	4	0	0	
Joint pain															
No	80	98	5	100	1.000	78	98	7	100	1.000	50	100	9	100	
Yes	2	2	0	0		2	3	0	0			0		0	
Body ache															
No	82	100	5	100		80	100	7	100		50	100	9	100	
Yes		0		0			0		0			0		0	
Cough															
No	79	96	5	100	1.000	77	96	7	100	1.000	48	96	9	100	1.000
Yes	3	4	0	0		3	4	0	0		2	4	0	0	
Diarrhoea															
No	81	99	5	100	1.000	79	99	7	100	1.000	49	98	9	100	1.000
Yes	1	1	0	0		1	1	0	0		1	2	0	0	
Headache															
No	79	96	5	100	1.000	77	96	7	100	1.000	49	98	9	100	1.000
Yes	3	4	0	0		3	4	0	0		1	2	0	0	
Nausea															
No	82	100	5	100		80	100	7	100		50	100	9	100	
Yes		0		0			0		0			0		0	
Vomiting															
No	81	99	5	100	1.000	79	99	7	100	1.000	49	98	9	100	1.000
Yes	1	1	0	0		1	1	0	0		1	2	0	0	
Sweat															
No	81	99	5	100	1.000	79	99	7	100	1.000	50	100	9	100	
Yes	1	1	0	0		1	1	0	0			0		0	
Chills and shivers															
No	80	98	5	100	1.000	78	98	7	100	1.000	49	98	9	100	1.000
Yes	2	2	0	0		2	3	0	0		1	2	0	0	
Weakness															
No	79	96	5	100	1.000	77	96	7	100	1.000	48	96	9	100	1.000
Yes	3	4	0	0		3	4	0	0		2	4	0	0	

RDT: Rapid diagnostic test; ITN: Insecticide treated nets; IRS: Indoor residual spraying

### *Plasmodium falciparum* positivity rate and association with clinical data

One-hundred and eighty-six (19.6%) and 200 (21.0%) of the 951 febrile participants had a *P. falciparum*-positive result by RDT and microscopy, respectively (Table 1). Positivity rate increased with age and with the reporting of joint pain, chills and shivers, headache, vomiting and weakness. In contrast, it was lower in patients with cough and nausea compared to those with other signs of malaria. The positivity rate among pregnant women at first ANC visit was 5.7% (5 out of 87 women) and 8.0% (7 out of 87) by RDT and microscopy, respectively (Table 2). None of the clinical variables tested were associated with positivity by RDT, microscopy or qPCR.

### RDT performance

Compared to microscopy (Table 3), RDT sensitivity was 89% (185/207), with a false negativity rate of 11% (22/207). RDT specificity was 99% (825/831) and the false positivity rate was < 1% (6/831). Among the 28 samples with discordant RDT and microscopy results, 21 (5 RDT-positive but microscopy-negative, and 16 RDT-negative but microscopy-positive) were available for molecular analysis to screen for *P. falciparum* DNA using qPCR. Three (60%) of the 5 samples found to be positive by RDT but negative by microscopy were negative by qPCR, while 2 (40%) of them were positive by qPCR. The 16 (100%) samples, which were positive by microscopy but negative by RDT, were also negative by qPCR. Among the 22 samples positive by microscopy and negative by the 18S *P. falciparum* qPCR, only one was positive for *Plasmodium* spp. (Ct = 39.8) [24]. Among the randomly selected 224 samples that were negative by RDT and microscopy, 14 (6.2%) were confirmed positive by qPCR. *Plasmodium falciparum* densities (as quantified by qPCR) were higher among RDT-positive infections (n = 72, geometric mean: 767.9 parasites/μL; SD: 2,938.2) than RDT-negative infections (n = 14, 2.1, SD: 2.9; p < 0.001). The 14 samples were tested for the presence of *hrp2/3* deletions using a multiplex qPCR assay that targets *pfhrp2*, *pfhrp3*, *pfldh*_ *HumanTuBB*_ [25]. The 14 samples were positive for the human gene (*HumTuBB*), but only 3 amplified *pfldh*. Among these 3 samples with enough parasitaemias for the detection of single copy parasite genes (*pfpldh*), all were positive for *hrp2* and *hrp3*.

**Table 3:** Concordance of diagnostic results between microscopy and RDT, and confirmation by qPCR targeting *Plasmodium falciparum* 18S rRNA

	All				Non-pregnant				Pregnant			
	Microscopy				Microscopy				Microscopy			
	Neg		Pos		Neg		Pos		Neg		Pos	
	n = 831		n = 207		n = 751		n = 200		n = 80		n = 7	
RDT	n	%	n	%	n	%	n	%	n	%	n	%
Neg	825	99	22	11	745	99	20	10	80	100	2	29
Pos	6	1	185	89	6	1	180	90	0	0	5	71

	PCR results				PCR results				PCR results			
	Neg		Pos		Neg		Pos		Neg		Pos	
	n = 235		n = 86		n = 185		n = 77		n = 50		n = 9	
	n	%	n	%	n	%	n	%	n	%	n	%
RDT−/MIC−	210	89	14	16	161	87	9	12	49	98	5	56
RDT−/MIC+	16	7	0	0	16	9	0	0	0	0	0	0
RDT+/MIC−	3	1	2	2	3	2	2	3	0	0	0	0
RDT+/MIC+	6	3	70	81	5	3	66	86	1	2	4	44

## Discussion

This study provides evidence of the absence of qPCR-positive, false-negative RDT results and therefore of *pfhrp2/3* deletions in *P. falciparum* isolates circulating in Monrovia (Liberia). Among the 1,038 individuals included in the study, only 22 had a negative RDT and a positive microscopy. Sixteen of these samples tested by qPCR confirmed the absence of *P. falciparum* DNA, therefore indicating a false positive result by microscopy. Results of this study suggest that *P. falciparum* parasites circulating in Monrovia do not yet carry *pfhrp2/hrp3* deletions and are, therefore, conveniently detectable using PfHRP2-based RDTs. However, continuous monitoring for the emergence of PfHRP2 deletions is needed to avoid RDT failures that could potentially compromise malaria control programmes in Liberia.

The prevalence of *P. falciparum* infections among individuals with fever or history of fever during the preceding week was 19.6% by RDT and 21.0% by microscopy. Among those individuals who were negative by both diagnostic tests, the prevalence of *P. falciparum* infection by qPCR was 5.3%, indicating a moderate level of low-density malaria infections, which are undetected among febrile individuals. The carriage of sub-patent infections might be higher among afebrile individuals, as observed in pregnant women at first ANC visit (9.2%), who tend to carry asymptomatic low-density infections [26]. Overall, the low malaria positivity rates in Monrovia compared to estimates from other African countries [27, 28] might be due to the relatively lower risk of malaria infection among the population residing in Monrovia compared to the rural areas in Liberia. Positivity rate is higher among individuals reporting joint pain, vomiting, chills and shivers and weakness. In contrast, cough and nausea were associated with lower malaria positivity rates, suggesting these clinical signs may appear to be resulted by other diseases such as respiratory infections. Positivity rate was also higher among older individuals, suggesting that occupational or motility factors may contribute to increased risk of exposure to malaria parasites in areas outside Monrovia with higher transmission.

The specificity of RDT, compared to microscopy, was high (99%), with most of the false-positive results being negative by qPCR, suggesting HRP2 persistence after a recently cleared *P. falciparum* infection [4]. False-negative results were higher, with 11% of the microscopic-positive subjects negative for RDT. This is below the overall estimate of 19.9% obtained from community-based malaria surveys in 19 sub-Saharan African countries [13]. Importantly, qPCR confirmed the absence of *P. falciparum* DNA in the 16 RDT-negative and microscopy-positive samples that were available for molecular testing. All these samples were also negative for other *Plasmodium* human species [24]. Only one of the 6 samples which were positive by both RDT and microscopy was positive for *Plasmodium* spp, therefore indicating a non-falciparum *Plasmodium* spp. that was missed by the first qPCR targeting *P. falciparum* 18S rRNA. Finally, among the 14 samples that were positive by qPCR but negative by RDT and microscopy, 3 only amplified *pfldh*, and among these 3 all were positive for *hrp2* and *hrp3* [25]. The lack of amplification of *pfldh* in the 11 samples is probably due to very low density infections which are still amplified by the 18S rRNA (multigene) qPCR but not by the single-gene qPCR (*pfldh*). Independent of the reason above, this study rules out the possibility of true (qPCR-confirmed) parasitaemic cases undetected by the RDT. This thus provides evidence that none of the parasite isolates collected in this study was potential carriers of *pfhrp2/hrp3* deletions.

This study has several limitations. First, a sub-set of dried blood spots (including 6 of the 22 which were collected from individuals with RDT-negative but microscopy-positive results) were not available for molecular testing. And second, the fees of consultation and malaria diagnostic tests in the institution of recruitment may have led to an under-representation of populations with low social-economic backgrounds who may be more prone to malaria infection.

## Conclusions

PfHRP2-based RDTs are efficacious in detecting the majority of the malaria parasites in the Monrovia area, with no evidence of *pfhrp2/3* deletions in this parasite population.

## Data Availability

The datasets generated during and/or analysed during the current study are not publicly available due to the agreements reached with the regulatory authorities of the country but are available from the corresponding author on reasonable request.

## Abbreviations

18 S rRNA: 18 Small ribonuclease ribonucleic acid
ANC: Antenatal Carre
pAldo: *Plasmodium* aldolase
PfHRP2*/3*: *Plasmodium falciparum* Histidine-rich protein 2 and 3
Pldh: *Plasmodium* lactate dehydrogenase
qPCR: Quantitative polymerase chain reaction
RDTs: Rapid diagnostic tests
SJCH: Saint Joseph’s Catholic Hospital
WHO: World Health Organization
